# Engineering spin-dependent catalysts: chiral covalent organic frameworks with tunable electroactivity for electrochemical oxygen evolution

**DOI:** 10.1093/nsr/nwae332

**Published:** 2024-09-16

**Authors:** Ziping Li, Yueyuan Xiao, Chao Jiang, Bang Hou, Yan Liu, Yong Cui

**Affiliations:** School of Chemistry and Chemical Engineering, Frontiers Science Center for Transformative Molecules and State Key Laboratory of Metal Matrix Composites, Shanghai Jiao Tong University, Shanghai 200240, China; School of Chemistry and Chemical Engineering, Frontiers Science Center for Transformative Molecules and State Key Laboratory of Metal Matrix Composites, Shanghai Jiao Tong University, Shanghai 200240, China; School of Chemistry and Chemical Engineering, Frontiers Science Center for Transformative Molecules and State Key Laboratory of Metal Matrix Composites, Shanghai Jiao Tong University, Shanghai 200240, China; School of Chemistry and Chemical Engineering, Frontiers Science Center for Transformative Molecules and State Key Laboratory of Metal Matrix Composites, Shanghai Jiao Tong University, Shanghai 200240, China; School of Chemistry and Chemical Engineering, Frontiers Science Center for Transformative Molecules and State Key Laboratory of Metal Matrix Composites, Shanghai Jiao Tong University, Shanghai 200240, China; School of Chemistry and Chemical Engineering, Frontiers Science Center for Transformative Molecules and State Key Laboratory of Metal Matrix Composites, Shanghai Jiao Tong University, Shanghai 200240, China

**Keywords:** 3D chiral COF, spin-dependent catalysts, CISS, electrochemical oxygen evolution

## Abstract

The chiral-induced spin selectivity (CISS) effect offers promising prospects for spintronics, yet designing chiral materials that enable efficient spin-polarized electron transport remains challenging. Here, we report the utility of covalent organic frameworks (COFs) in manipulating electron spin for spin-dependent catalysis via CISS. This enables us to design and synthesize three three-dimensional chiral COFs (CCOFs) with tunable electroactivity and spin-electron conductivity through imine condensations of enantiopure 1,1′-binaphthol-derived tetraaldehyde and tetraamines derived from 1,4-benzenediamine, pyrene, or tetrathiafulvalene skeletons. The CISS effect of CCOFs is verified by magnetic conductive atomic force microscopy. Compared with their achiral analogs, these CCOFs serve as efficient spin filters, reducing the overpotential of oxygen evolution and improving the Tafel slope. Particularly, the diarylamine-based CCOF showed a low overpotential of 430 mV (vs reversible hydrogen electrode) at 10 mA cm^−2^ with long-term stability comparable to the commercial RuO_2_. This enhanced spin-dependent OER activity stems from its excellent redox-activity, good electron conductivity and effective suppression effect on the formation of H_2_O_2_ byproducts.

## Teaser

This work represents the pioneering utilization of COFs as spin-dependent catalysts for electrochemical OER, showcasing the immense potential of CCOFs for electron spin polarization and their application in catalysis.

## INTRODUCTION

Since the discovery of the chiral-induced spin selectivity (CISS) phenomenon in double-stranded DNA [1], where spin electrons can be extracted and utilized by chiral compounds with a preferred spin orientation, this field has rapidly developed [2–4]. The CISS effect offers an innovative approach to spintronics and quantum-based devices [5,6], particularly for chiral materials, holding great potential for applications in chiral sensing [7], circularly polarized luminescence (CPL) [8], spin-dependent catalysis [9,10], and magnetoresistance [11]. Recently, a range of chiral organic assemblies and their hybrid materials have been devised for CISS, encompassing organic polymers [12], metal-organic frameworks (MOFs) [13], overcrowded alkenes [14], and organic-inorganic perovskites [15]. They can act as spin filters to address weak spin-orbit interaction (SOI) and exchange interaction [16–18], while also offering desirable advantages such as flexibility and compatibility with biological systems [19,20]. Nonetheless, designing chiral materials capable of enabling efficient spin-polarized electron transport and effective spin manipulation remains a persistent challenge [21,22]. Here, we illustrate that covalent organic frameworks (COFs) can serve as a platform to manipulate CISS for spin-dependent catalysis.

COFs are a relatively new class of crystalline organic materials constructed by organic monomers via dynamic covalent bonds [23–25]. Due to their porous and modular nature, COFs have been extensively studied in gas storage [26], separation [27], biomedicine [28], and heterogeneous catalysis [29]. COFs provide a promising avenue for engineering crystalline chiral organic materials by strategically selecting enantiopure building blocks [30], templates [31], or additives [32]. Compared to chiral MOFs that are highly structurally similar to them [33], chiral COFs (CCOFs) are generally more chemically robust because of their stronger covalent linkages, and thus have been broadly employed as durable materials for enantioselective sensing [34], enantioseparation [35], heterogeneous asymmetric catalysts [36,37]. However, research on CISS within COFs remains largely unexplored [38]. Specifically, there have been no reported studies on the role of spin electrons in catalysis within the realm of COFs, despite the potential of the CISS effect to offer a viable approach toward this goal [38,39]. One potential approach for spin-dependent catalysis involves modulating the spin electrons of targeted catalysts to generate spin-polarized free radical intermediates, which can subsequently engage in catalytic reactions [40,41]. Exploring how to tune the electroactivity and spin-electron conductivity is the key to achieve highly efficient spin-dependent catalysts [10,42].

**Scheme 1.**
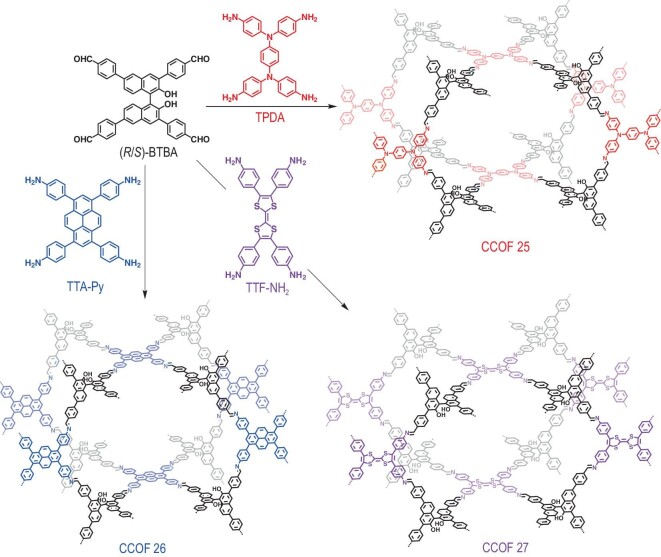
Syntheses of CCOFs **25–27**.

In this work, we report the engineering of spin-dependent CCOF catalysts for electrochemical oxygen evolution reaction (OER) by customizing redox activity and electronic properties of building blocks. We synthesized and structurally characterized three electroactive three-dimensional (3D) CCOFs by introducing optically pure 1,1′-binaphthol (BINOL) as chiral site and three redox-active organic monomers TPDA, TTA-Py and TTF-NH_2_ (Scheme 1). By deploying pre-designed monomers with 1,4-benzenediamine, pyrene, or tetrathiafulvalene cores, we can finely tune the electroactivity and spin-electron conductivity of CCOFs. These materials serve as highly efficient spin filters, reducing the overpotential of oxygen evolution and improving the Tafel slope compared to the achiral analogs. The CISS effect of CCOFs was observed through magnetic conductive atomic force microscopy (mc-AFM) measurements. Notably, the diarylamine-based CCOF exhibited a low overpotential (*η*) of 430 mV (vs reversible hydrogen electrode (RHE)) at 10 mA cm^−2^ with excellent long-term stability, close to commercial RuO_2_ [29,43]. This spin-dependent OER activity stemmed from good electron conductivity, excellent diarylamine redox-active cores, and effective suppression of hydrogen peroxide byproducts.

## RESULTS AND DISCUSSION

### Synthesis and characterization

Using enantiopure (*R*)- or (*S*)-2,2′-dihydroxy-1,1′-binaphthyl-3,3′,6,6′-tetrakis(4-benzaldehyde) (**BTBA**) as a chiral monomer, electroactive frameworks were constructed by incorporating *N*,*N*,*N*′, *N*′-tetrakis(4-aminophenyl)-1,4-benzenediamine (**TPDA**), 1,3,6,8-tetrakis(4-aminophenyl)pyrene (**TTA-Py**) or 4,4′,4′′,4′′′-([2,2′-bi(1,3-dithiolylidene)]-4,4′,5,5′-tetrayl)tetraaniline (**TTF-NH**_2_). As illustrated in Scheme 1, CCOFs **25**–**27** were prepared under solvothermal conditions using (*R*)- or (*S*)-**BTBA** and **tetraamine** (1 : 1) in a mixture of chloroform (CHCl_3_) and *o*-dichlorobenzene (*o*-DCB) (1/9, v/v), catalyzed by acetic acid at 100°C for 72 h. The reactions yielded brown, yellow and dark-red polycrystalline powders in 84%, 91%, and 79% yields, respectively. Related racemic frameworks were prepared following the same procedure using racemic **BTBA** (*Rac*-**BTBA**).

Initially, the as-prepared CCOFs were structurally characterized by Fourier transform infrared (FT-IR) spectroscopy, ^13^C cross-polarization magic-angle-spinning (CP-MAS) NMR and circular dichroism (CD) spectra. For FT-IR spectra, the aldehyde peak at 1699 cm^−1^ was greatly reduced, and the characteristic C=N stretching bands were obtained at 1621 cm^−1^ for **25**, 1619 cm^−1^ for **26**, and 1622 cm^−1^ for **27** (Fig. S1). Additionally, the successful formation of amine linkage was confirmed by ^13^C CP/MAS NMR. The signal at ∼155.9 ppm can be attributed to the carbon atom at –C=N– in the spectrum of **25**, and similar signals for **26** and **27** were present at ∼156.9 and 158.5 ppm (Fig. S2), respectively. The three pairs of chiral COFs prepared from (*R*)- and (*S*)-**BTBA** are mirror images of each other in the CD spectra, indicating their enantiomeric nature (Fig. S3).

### Crystal structure

**Figure 1.**
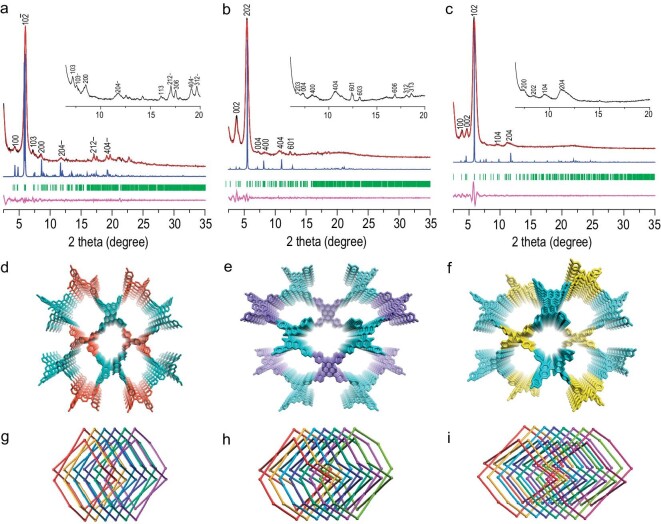
(a–c) PXRD profiles of CCOFs **25**–**27**. Experimental curve (black line), Pawley refined (red line), simulated curve (blue line), *Bragg* positions (green line) and their difference (pink line). (d–f) The stick model for **25**–**27**. (g–i) Interpenetration of 6-, 7- and 8-fold **pts** nets in **25**–**27**, respectively.

**Figure 2.**
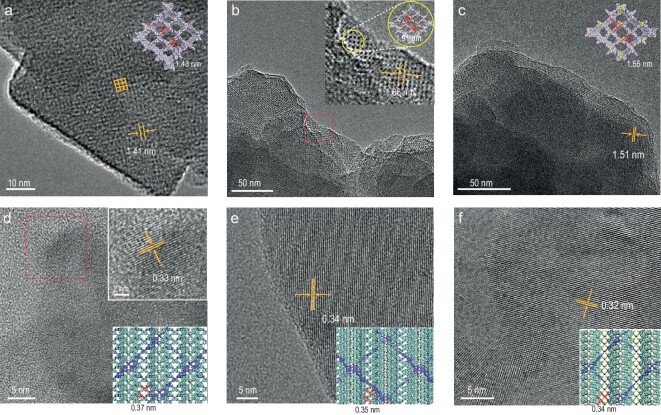
(a) HR-TEM image of CCOF **25** (inset: the space-filling model of CCOF **25**). (b) HR-TEM image of CCOF **26** (inset: the enlarged TEM image of red square area, where the rhombus lattice is like the space-filling model, and the distance of two adjacent lattice fringes is 1.86 nm, which is close to that of the model). (c) HR-TEM image of CCOF **27** (inset: the space-filling model of CCOF **27**). HR-TEM images for the lattice fringes generated by the interpenetrated **pts** network along the *b*-axis and the corresponding stick model as inserted graph: (d) CCOF **25**, (e) CCOF **26**, (f) CCOF **27**.

The crystalline structures of CCOFs were determined by powder X-ray diffraction (PXRD) analysis with Cu Kα radiation. The experimental PXRD pattern for CCOF **25** exhibited some peaks at 4.27° ± 0.02°, 5.99° ± 0.02°, 7.10° ± 0.04°, 7.58° ± 0.03°, 8.44° ± 0.04°, 11.82° ± 0.05°, 16.09° ± 0.04°, 17.08° ± 0.05°, 17.52° ± 0.04°, 19.11° ± 0.03°, and 19.68° ± 0.05°, which were assigned to (100), (10$\overline{2}$), (103), (10$\overline{3}$), (200), (20$\overline{4}$), (113), (21$\overline{2}$), (306), (40$\overline{4}$), and (31$\overline{2}$) plane facets, respectively (Fig. 1a). Similarly, CCOF **26** exhibited some peaks at 3.66° ± 0.02°, 5.44° ± 0.01°, 6.75° ± 0.03°, 7.30° ± 0.02°, 8.31° ± 0.04°, 10.81° ± 0.04°, 12.42° ± 0.05°, 13.21° ± 0.04°, 16.89° ± 0.05°, 18.13° ± 0.06°, and 18.61° ± 0.06°, corresponding to the (002), (202), (203), (004), (400), (404), (601), (603), (606), (312), and (313) plane facets, respectively (Fig. 1b). For CCOF **27**, some peaks appeared at 3.76° ± 0.03°, 4.59° ± 0.04°, 5.81° ± 0.03°, 7.46° ± 0.03°, 8.42° ± 0.04°, 9.56° ± 0.03°, and 11.48° ± 0.07°, which were assigned to the (100), (002), (102), (200), (202), (104), and (204) plane facets, respectively (Fig. 1c). The structure models were built by using the Materials Studio software package. Considering the geometry of monomers and the stoichiometric ratio, a few possible nets (e.g. **pts, ptt, pth, pti, dia**, etc.) for three CCOFs and two-dimensional (2D) **sql** net were constructed according to Reticular Chemistry Structure Resource (RCSR) (Figs S4–S14) [44]. After considering these possible interpenetrated nets with different space groups, the detailed simulation clearly suggest that CCOFs **25** and **27** are proposed to adopt 6-fold and 8-fold interpenetrated **pts** topology with the *P*2 space group, and the PXRD of CCOF **26** agrees with 7-fold interpenetrated **pts** topology with the *P*2_1_ space group (Fig. 1d–i). A full profile Pawley refinement was performed to obtain the unit cell parameters (a = 20.58 Å, b = 6.26 Å, c = 43.89 Å, α = γ = 90°, β = 88.7°; *R*_wp_ = 4.35%, *R*_p_ = 3.15% for CCOF **25**; a = 43.32 Å, b = 5.37 Å, c = 47.67 Å, α = β = γ = 90°, *R*_wp_ = 4.59%, *R*_p_ = 3.27% for CCOF **26**; a = 23.89 Å, b = 5.59 Å, c = 38.87 Å, α = β = γ = 90°, *R*_wp_ = 5.59%, *R*_p_ = 4.47% for CCOF **27**), which led to satisfactorily low residual values and acceptable profile differences. In addition, the PXRD curves of the three racemic COFs were also obtained, which were similar to their corresponding PXRD patterns of CCOFs (Figs S15–S17). The simulation results indicate that all racemic COFs adopt the same fold interpenetrated **pts** topology of *P*1 space group with their corresponding CCOFs. The scanning electron microscopy images indicate all three chiral microcrystals are prone to aggregate into irregular spherical particles under solvent thermal conditions (Fig. S18). The high-resolution transmission electron microscopy (HR-TEM) image of CCOF **25** revealed a highly ordered porous network with rhombic pores (marked with an orange grid, Fig. 2a). The magnified TEM image showed that the distance between two adjacent pores is ∼1.41 nm for CCOF **25** (Fig. S19b), closely aligning with the *d*-spacing of 1.43 nm between two adjacent edges in the simulated cell (Fig. 2a, inset). In the case of **26**, the distinct rhombic skeleton was directly observable and bore a resemblance to the model (Fig. 2b). The measured distance of 1.86 nm in the HR-TEM image was consistent with the size of the rhombic lattice, ∼1.91 nm. Additionally, for **27**, similar lattice fringes were also observed, which is close to its model (Fig. 2c). In addition, lattice fringes generated by the interpenetrated **pts** crystalline network along the *b*-axis were observable in the HR-TEM image, with fringe spacing closely matching the corresponding model's prediction of 0.37 nm for CCOF **25**, 0.35 nm for CCOF **26**, and 0.34 nm for CCOF **27** (Fig. 2d–f).

### Porosity and stability

The permanent porosity of CCOFs was investigated by the N_2_ sorption isotherms at 77 K. As shown in Fig. S20, all three CCOFs exhibited a sharp step at a relative low pressure (*P*/*P*_0_ < 0.02), indicating their microporous nature. The Brunauer–Emmett–Teller (BET) surface areas were measured as 739 m^2^ g^−1^ for (*R*)-**25**, 1197 m^2^ g^−1^ for (*R*)-**26**, and 657 m^2^ g^−1^ for (*R*)-**27**. The corresponding total pore volumes at *P*/*P*_0_ = 0.99 were calculated to be 0.40 cm^3^ g^−1^, 0.64 cm^3^ g^−1^, and 0.34 cm^3^ g^−1^, respectively. The pore size distribution of the three CCOFs, calculated using nonlocal density functional theory (NLDFT), revealed main pore widths of ∼8 Å and 10 Å (Fig. S20), which align with the values from the simulated structures (Fig. S21). Similar BET results were observed for the corresponding racemic COFs (Fig. S20).

Thermogravimetric analysis (TGA) revealed that the three CCOFs exhibit good thermal stability, with (*R*)-**25**, (*R*)-**26**, and (*R*)-**27** remaining stable up to 481°C, 520°C, and 419°C, respectively (Fig. S22). Additionally, the chemical stability of the prepared CCOFs was assessed by examining the PXRD patterns and N_2_ sorption isotherms after immersing the samples in various organic solvents, boiling water, and aqueous HCl (3.0 M) and NaOH (3.0 M) solutions at room temperature for 3 days. The treated samples exhibited only minor reductions in PXRD peak intensities and a slight decrease in BET surface areas, indicating that all COFs possess good chemical solvent stability, which is advantageous for their application as catalysts in acidic or basic environments (Figs S20 and S23).

### The CISS effect

**Figure 3.**
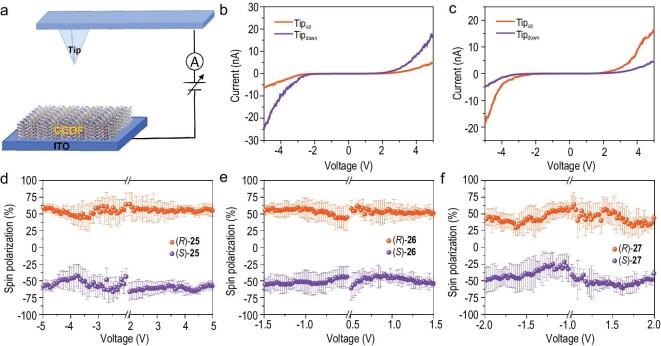
(a) Schematic illustration of mc-AFM measurement setup for CCOFs. The averaged *I*–*V* curves of charge transport through (b) (*R*)-**25** and (c) (*S*)-**25**. The tip is magnetized upward (tip upward) and downward (tip downward). The spin polarized curves of (d) (*R*)- and (*S*)-**25**, (e) (*R*)- and (*S*)-**26**, (f) (*R*)- and (*S*)-**27**.

To investigate the CISS effect in these CCOFs, we recorded the spin-polarized charge transport signal by mc-AFM (Fig. 3a). The potential is applied between the CCOF-loaded indium tin oxide (ITO) substrate and magnetized ferromagnetic tip, which has two magnetism directions (upward and downward) with respect to the substrate after magnetizing by a permanent magnet. The current–voltage (*I–V*) curves, corresponding to various magnetization directions, were measured at least 30 times from different positions, with the raw data being presented in Figs S24–S26. For (*R*)-**25**, the averaged current curves showed remarkable difference for two magnetization directions. The current values under upward magnetization were much lower than that under downward magnetization at the potential windows of −5 to 5 V (Fig. 3b). This trend was completely opposite for (*S*)-**25** (Fig. 3c), where the current values under upward magnetization were much higher than that under downward magnetization. The mc-AFM results indicate a pronounced spin-polarized charge transport in CCOFs, namely electron spins can be polarized when transporting through **25**, which is attributed to the CISS effect. The CISS phenomenon was also observed in **26** and **27** (Figs S25 and S26). The spin polarization (*P*) can be used to quantify the anisotropy of polarized current, which is determined by the equation *P* = (*I*_down_ − *I*_up_)/(*I*_up_ + *I*_down_) × 100%, Where *I*_up_ and *I*_down_ represent the current values with magnetization upward and downward at a specific applied potential, respectively. The *P* value of **25** systems was calculated to be 57% ± 5% (Fig. 3d). For **26** and **27**, the *P* values were determined to be 52% ± 4% and 41% ± 4%, respectively (Fig. 3e, f).

### The OER activity

**Figure 4.**
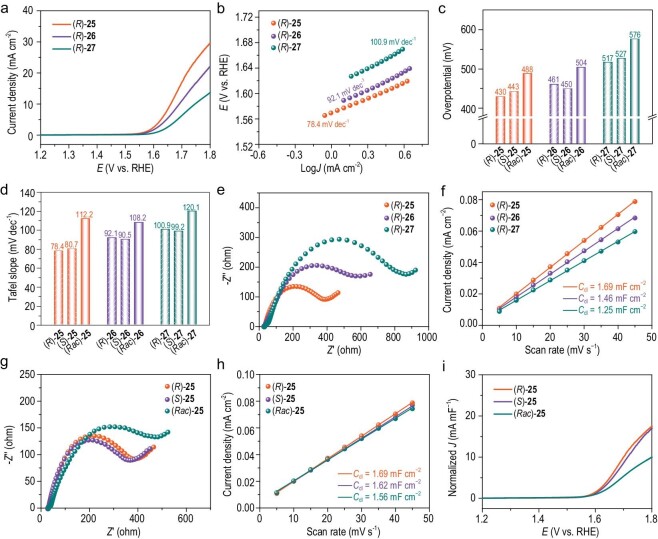
(a) Polarization curves of the three (*R*)-CCOFs with 90% *iR* compensation. (b) Tafel plots of the three (*R*)-CCOFs. (c) Overpotential of all the chiral and racemic COFs. (d) Tafel slope of all the chiral and racemic COFs. (e) EIS Nyquist plots of the three (*R*)-CCOFs. (f) Fitted relationships between capacitive current and scan rate, and corresponding *C*_dl_ values for the three (*R*)-CCOFs. (g) EIS Nyquist plots of (*R*)- and (*S*)-**25**, and (*Rac*)-**25**. (h) Fitted relationships between capacitive current and scan rate, and corresponding *C*_dl_ values for (*R*)- and (*S*)-**25**, and (*Rac*)-**25**. (i) Normalized polarization curves against *C*_dl_ value of (*R*)- and (*S*)-**25**, and (*Rac*)-**25**.

To determine the electrocatalytic activities of these metal-free COFs, linear sweep voltammetry (LSV) curves were recorded in O_2_-saturated KOH solution (1.0 M) at room temperature after reaching an electrochemical stable state through preprocessing. (*R*)-**25** showed an overpotential (*η*) of 430 mV (vs RHE) at 10 mA cm^−2^, which is lower than that of Co-TAPP-COF (473 mV) [45] and PCN-226(Co) (445 mV) [46], and comparable to the value of commercial RuO_2_ (430 mV) [29] and some reported metal-based porous materials [47,48] (Fig. 4a and Table S7). The overpotentials (*η*_10_) of (*R*)-**26** and (*R*)-**27** were 461 mV and 517 mV, respectively, both of which are higher than that of (*R*)-**25**. The corresponding Tafel slope was calculated to be 78.4 mV dec^−1^ for (*R*)-**25**, 92.1 mV dec^−1^ for (*R*)-**26**, and 100.9 mV dec^−1^ for (*R*)-**27** (Fig. 4b). In addition, the chirality effect on OER activity was also investigated. As illustrated in Fig. 4c and Fig. S27, (*S*)-**25** (*η*_10_ = 443 mV) exhibited a similar overpotential with (*R*)-**25**, which is obviously lower than that of racemic COF **25** ((*Rac*)-**25**) (*η*_10_ = 488 mV). The OER activity enhancement phenomenon by chirality was also observed from the LSV plots of CCOF **26** and **27**. The *η*_10_ of (*S*)-**26** and (*S*)-**27** were 450 mV and 527 mV, which are lower than those of (*Rac*)-**26** and (*Rac*)-**27**. The Tafel slope was evaluated to be 80.7 mV dec^−1^ for (*S*)-**25**, 90.5 mV dec^−1^ for (*S*)-**26**, and 99.2 mV dec^−1^ for (*S*)-**27**, respectively (Fig. 4d and Fig. S28). And the corresponding Tafel slope values of achiral COFs were increased to 112.2 mV dec^−1^ for (*Rac*)-**25**, 108.2 mV dec^−1^ for (*Rac*)-**26**, and 120.1 mV dec^−1^ for (*Rac*)-**27**, respectively. Furthermore, we investigated the long-term performance of (*R*)-**25** toward OER by chronoamperometry (Fig. S29). The current density was kept at ∼17.6 mA cm^−2^ for 24 h without obvious decline, and the used sample still displayed a similar overpotential (*η*_10_ = 439 mV), close to that of the original sample. Moreover, we conducted a cyclic voltammetry (CV) stability measurement, the CV curves did not change significantly after reaching a steady state (Fig. S30a). Also there was no significant decrease in crystallinity (Fig. S30b) for the used sample, and it well retained the original skeleton connectivity (Fig. S30c) and morphology (Fig. S30e). These results indicate that (*R*)-**25** is a stable electrocatalyst for OER.

To understand the OER activity difference for the three CCOFs, electrochemical impedance spectroscopy (EIS) and electrochemically active surface area (ECSA) measurements were conducted. (*R*)-**25** displayed a smaller circle diameter than that of (*R*)-**26** and (*R*)-**27**, indicating a faster interfacial charge transfer process in (*R*)-**25** (Fig. 4e). The superior conductivity of (*R*)-**25** was also confirmed by broadband dielectric spectroscopy (Fig. S31). Since ECSA is proportional to the double-layer capacitance (*C*_dl_) and the same condition was employed, we can estimate the active surface area of catalysts by *C*_dl_, which can be obtained by fitting the current density of the CV curve in the non-Faradaic region against scan rate (Fig. S32) [49]. (*R*)-**25** showed a larger *C*_dl_ value of 1.69 mF cm^−2^ than (*R*)-**26** (1.46 mF cm^−2^) and (*R*)-**27** (1.25 mF cm^−2^) (Fig. 4f). It indicates more electrochemical active sites for OER in (*R*)-**25**. To eliminate the effects caused by different porosity in catalytic performance, we normalized current density against BET surface areas and *C*_dl_ against BET surface areas. Figs S33 and S34 showed that (*R*)-**25** exhibits the highest OER activity and largest *C*_dl_ due to its diarylamine units, which act as electron donors and redox-active cores, facilitating intramolecular electron transport and expanding the electrochemically active surface areas [43]. Additionally, CV measurements were conducted, revealing oxidation potentials (vs. saturated calomel electrode (SCE)) of 0.59 V for (*R*)-**25**, 0.62 V for (*R*)-**26**, 0.68 V for (*R*)-**27**, respectively (Fig. S35). Thus, (*R*)-**25** exhibits the lowest oxidation potential among the three CCOFs, enabling easy oxidation to the activated state and facilitating participation in the electrocatalytic oxidation reaction, consistent with the LSV result [50]. Furthermore, we synthesized three amorphous chiral analogs of **25–27** and tested their LSV curves. The result showed that among the three amorphous materials, the amorphous (*R*)-**25** consistently exhibited superior OER performance (Fig. S36), albeit inferior to the crystalline (*R*)-**25**.

We explored the origin of enhanced OER activity by chirality through EIS and ECSA. The EIS results indicated that (*R*)-**25** and (*S*)-**25** exhibited lower resistance compared to (*Rac*)-**25**, and a similar trend was also observed in **26** and **27**, suggesting that chiral COFs facilitate faster interfacial charge transfer processes than their corresponding racemic COFs (Fig. 4g and Fig. S37) [41]. Moreover, we compared the ECSA of CCOFs with those of the achiral COFs. The *C*_dl_ values of (*R*)-**25**, (*S*)-**25**, and (*Rac*)-**25** were fitted to be 1.69, 1.62, and 1.56 mF cm^−2^, indicating approximately identical ECSA values between the chiral and achiral COFs (Fig. 4h). It suggested that they possess a comparable quantity of electroactive sites. However, after normalizing current density against *C*_dl_ values, (*R*)-**25** and (*S*)-**25** exhibited increased intrinsic OER activities compared with (*Rac*)-**25**, which revealed a chirality-enhanced OER activity (Fig. 4i) [41]. Similar chirality-improved OER behavior also occurred in **26** and **27** (Fig. S41). While the *C*_dl_ results suggested that the number of active sites is nearly identical in both chiral and achiral COFs, the CCOFs exhibit higher OER activity than their achiral counterparts, attributed to faster intermediate kinetics and more efficient interfacial charge transfer. In addition, the chirality-enhanced oxygen evolution activity was also evident in the LSV results of amorphous chiral and achiral COFs (Fig. S42).

**Figure 5.**
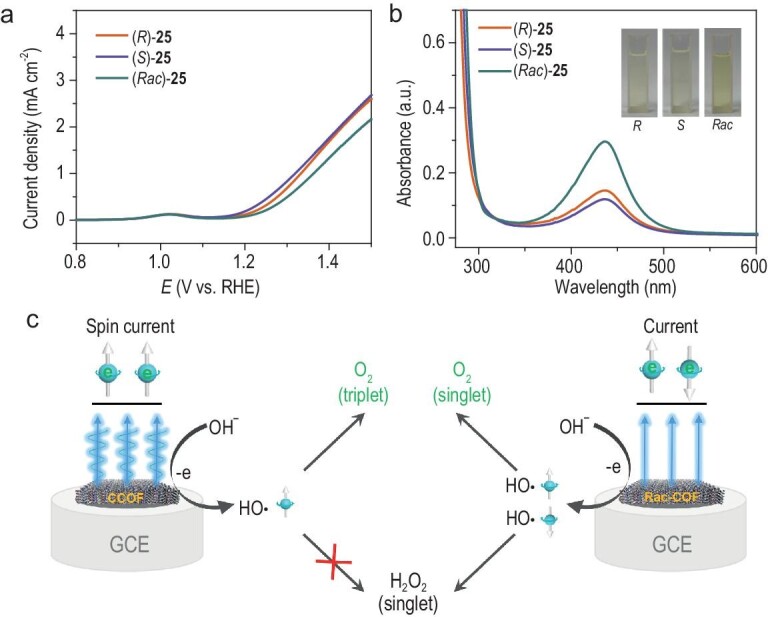
(a) The LSV plots of (*R*)- and (*S*)-**25**, and (*Rac*)-**25** in 0.1 M Na_2_SO_4_. (b) H_2_O_2_ detection results through spectrophotometry after bulk electrolysis for (*R*)- and (*S*)-**25**, and (*Rac*)-**25** in 0.1 M Na_2_SO_4_ solution at OER potential. In the chiral **25** system, only a portion of the spin electrons are polarized, and the hydroxyl radicals produced react with those having opposite spin directions to form H₂O₂. This results in a significantly lower amount of H₂O₂ compared to the racemic **25** system, where no spin polarization occurs. (c) The mechanism of spin-polarized oxygen evolution in chiral and achiral COFs.

Furthermore, the phenomenon of chirality-enhanced electrocatalytic activity can also be explained through the reaction mechanism. In previously reported studies, it was suggested that the CISS process influences the spin of electrons at the catalyst surface, which in turn affects the reaction pathway, thereby enhancing oxygen evolution [9,10,41]. The chirality-enhanced OER activity could be related to the suppression of H_2_O_2_ formation, which generated concomitantly as inevitable intermediates/byproducts during OER. To demonstrate this point, we conducted the LSV tests and the bulk electrolysis (BE) with chronoamperometry in 0.1 M Na_2_SO_4_ solution (pH 6.55), due to the unsuitability of stabilizing and detecting H_2_O_2_ in strongly alkaline systems. As shown in Fig. 5a and Fig. S43, the chirality-enhanced OER activities of CCOFs were observed in 0.1 M Na_2_SO_4_ solution, which is similar to the case in 1.0 M KOH. In addition, we detected H_2_O_2_ in the electrolyte after consuming an identical charge by a ultraviolet-visible (UV-vis) spectrometer using *o*-tolidine as the redox indicator. After a long period of OER catalyzed by (*Rac*)-**25**, a significant characteristic absorption peak of the produced H_2_O_2_ at ∼437 nm was observed in the reaction mixture with *o*-tolidine, which is higher than that of (*R*)-**25** and (*S*)-**25** systems (Fig. 5b). A similar behavior was also observed in **26** and **27** systems (Fig. S44). The formation of H_2_O_2_ byproducts relies on whether the electron spins of *OH intermediate are parallel or antiparallel, with the antiparallel configuration leading to the formation of H_2_O_2_ (Fig. 5c) [9]. Although racemic COFs can produce oxygen, the occurrence of the H_2_O_2_ pathway inhibits the oxygen evolution pathway. The CCOF film on a working electrode can create spin-polarized reaction intermediates and reduce *OH intermediates with antiparallel spin electron during electrocatalysis by spin filtering the anodic current, thus suppressing the H_2_O_2_ pathway and improving the efficiency of OER. Therefore, the chirality-enhanced effect of CCOFs was attributed to the improved electrocatalytic selectivity of O_2_ by CISS.

## CONCLUSIONS

In conclusion, we have successfully designed and synthesized three 3D CCOFs with **pts** topology, allowing for the precise tuning of electroactivity and spin-electron conductivity through the utilization of pre-designed monomers with 1,4-benzenediamine, pyrene, or tetrathiafulvalene cores. mc-AFM revealed that significant CISS effects in these CCOFs, with spin polarization ratios ranging from 41% to 57%. Compared to their achiral counterparts, these CCOFs serve as efficient spin filters, leading to reduced overpotential for OER and improved Tafel slope. Specifically, (*R*)-**25**, based on diarylamine, exhibited a low overpotential (*η*_10_) of 430 mV (vs RHE), demonstrating long-term stability close to commercial RuO_2_. The observed spin-dependent OER behavior within CCOFs can be attributed to their good electron conductivity, excellent diarylamine redox activity, and efficient suppression of hydrogen peroxide byproducts. To the best of our knowledge, this study represents the pioneering utilization of COFs as spin-dependent catalysts [30,38], showcasing the immense potential of CCOFs for electron spin polarization and their application in catalysis, which will promote the crafting of more spintronic organic polymers.

## Supplementary Material

nwae332_Supplemental_Files
